# 4′-Ferrocenyl-1′-methylacenapthylene-1-spiro-2′-pyrrolidine-3′-spiro-2′′-indane-2,1′′,3′′(1*H*)-trione

**DOI:** 10.1107/S1600536809049629

**Published:** 2009-11-25

**Authors:** B. Gunasekaran, S. Kathiravan, R. Raghunathan, V. Manivannan

**Affiliations:** a Department of Physics, AMET University, Kanathur, Chennai 603 112, India; b Department of Organic Chemistry, University of Madras, Guindy Campus, Chennai 600 025, India; c Department of Research and Development, PRIST University, Vallam, Thanjavur 613 403, Tamil Nadu, India

## Abstract

In the title compound, [Fe(C_5_H_5_)(C_29_H_20_NO_3_)], the acenaphthyl­ene ring system makes a dihedral angle of 83.77 (3)° with the indane-1,3-dione ring system. The central pyrrolidine ring exhibits a twist conformation. In the crystal, mol­ecules are linked by a weak inter­molecular C—H⋯O inter­action into a chain along the *b* axis. Two weak intra­molecular C—H⋯O inter­actions are also present.

## Related literature

For the biological activity of ferrocene derivatives, see: Biot *et al.* (2004 ▶); Fouda *et al.* (2007 ▶); Jaouen *et al.* (2004 ▶); Johnson & Sames (2000 ▶). For related structures, see: Stalin Elanchezhian *et al.* (2008 ▶); Kamala *et al.* (2009 ▶). For graph-set notation, see: Bernstein *et al.* (1995 ▶).
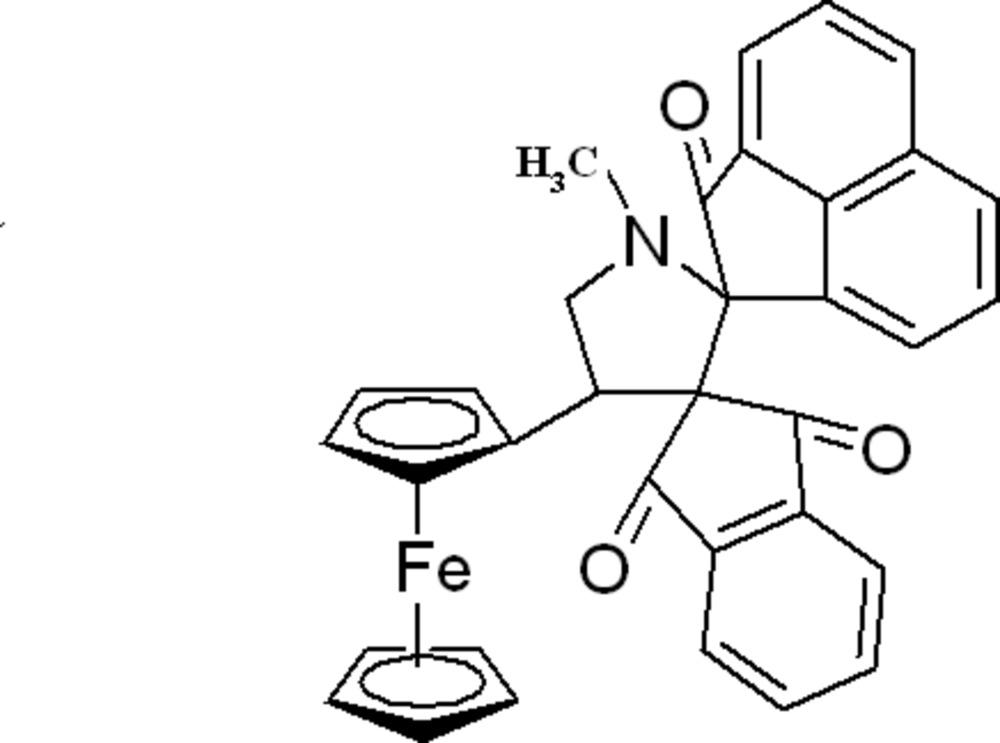


## Experimental

### 

#### Crystal data

[Fe(C_5_H_5_)(C_29_H_20_NO_3_)]
*M* *_r_* = 551.40
Monoclinic, 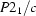

*a* = 12.5511 (10) Å
*b* = 10.8633 (9) Å
*c* = 19.2099 (16) Å
β = 103.432 (2)°
*V* = 2547.6 (4) Å^3^
*Z* = 4
Mo *K*α radiation
μ = 0.63 mm^−1^
*T* = 293 K
0.25 × 0.20 × 0.15 mm

#### Data collection

Bruker Kappa APEXII CCD diffractometer
Absorption correction: multi-scan (**SADABS**; Sheldrick, 1996 ▶) *T*_min_ = 0.860, *T*_max_ = 0.911
30734 measured reflections
6771 independent reflections
5179 reflections with *I* > 2σ(*I*)
*R*_int_ = 0.031

#### Refinement

*R*[*F* ^2^ > 2σ(*F* ^2^)] = 0.040
*wR*(*F* ^2^) = 0.124
*S* = 1.02
6771 reflections
353 parameters
5 restraints
H-atom parameters constrained
Δρ_max_ = 0.49 e Å^−3^
Δρ_min_ = −0.43 e Å^−3^

### 

Data collection: *APEX2* (Bruker, 2004 ▶); cell refinement: *SAINT* (Bruker, 2004 ▶); data reduction: *SAINT*; program(s) used to solve structure: *SHELXS97* (Sheldrick, 2008 ▶); program(s) used to refine structure: *SHELXL97* (Sheldrick, 2008 ▶); molecular graphics: *PLATON* (Spek, 2009 ▶); software used to prepare material for publication: *SHELXL97*.

## Supplementary Material

Crystal structure: contains datablocks global, I. DOI: 10.1107/S1600536809049629/is2493sup1.cif

Structure factors: contains datablocks I. DOI: 10.1107/S1600536809049629/is2493Isup2.hkl

Additional supplementary materials:  crystallographic information; 3D view; checkCIF report

## Figures and Tables

**Table 1:** Hydrogen-bond geometry (Å, °)

*D*—H⋯*A*	*D*—H	H⋯*A*	*D*⋯*A*	*D*—H⋯*A*
C3—H3⋯O3	0.98	2.48	3.045 (2)	116
C13—H13⋯O2	0.93	2.53	3.226 (2)	132
C8—H8⋯O2^i^	0.93	2.56	3.465 (6)	164

Symmetry code: (i) 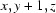.

**Fig. 1.**
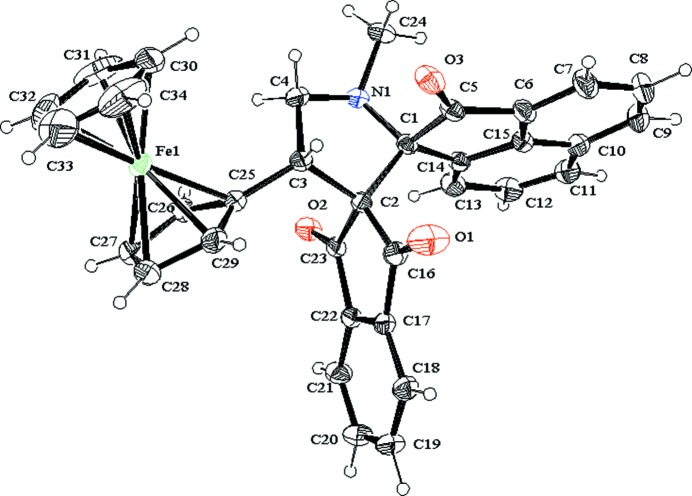
The molecular structure of (I), with atom labels and 30% probability displacement ellipsoids for non-H atoms.

**Fig. 2.**
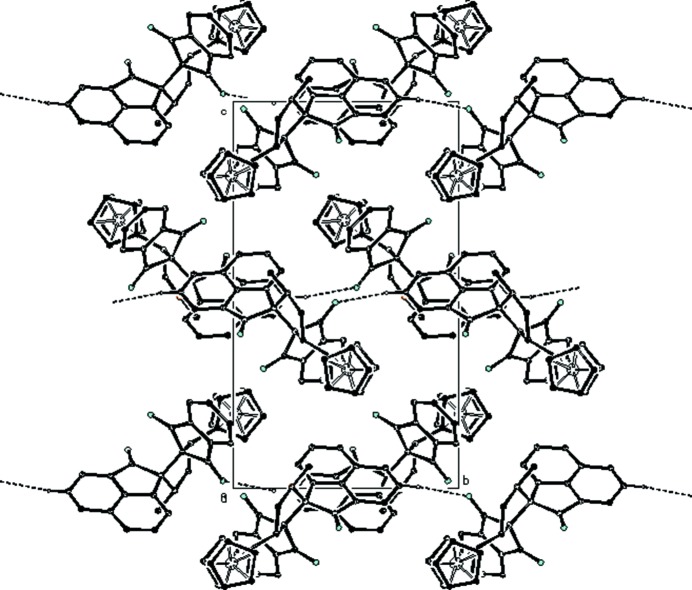
The packing of (I), viewed down the a axis. Hydrogen bonds are shown as dashed lines.

## supplementary crystallographic information

### Comment

Metallocenes are known to exhibit a wide range of biological activity. Among them ferrocenyl compounds display interesting antibacterial (Fouda *et al.*, 2007), antitumor (Jaouen *et al.*, 2004), antimalarial and antifungal (Biot *et al.*, 2004) activities. In addition, transition metal complexes derived from ferrocene have attracted great interest due to their applications as precursors for the synthesis of organic as well as organometallic compounds (Johnson & Sames, 2000).

The geometric parameters of the title compound (Fig. 1) agree well with reported similar structures (Stalin Elanchezhian *et al.*, 2008; Kamala *et al.*, 2009). The mean plane of acenaphthylene ring [C1/C5—C14] makes the dihedral angle of 83.77 (3)° with the indanedione ring [C2/C16—C23]. The sum of bond angles around N1 [339.72 (15)°] indicates *sp^3^* hybridization. The Fe1—*Cg*4 and Fe1—*Cg*5 distances are 1.6488 (3) and 1.6573 (3) Å, respectively, and the *Cg*4—Fe1—*Cg*5 angle is 177.70 (4)°, where *Cg*4 and *Cg*5 are the centroids of the substituted and unsubstituted cyclopentadienyl (Cp) rings, respectively. The small dihedral angle of 0.63 (11)° between the unsubstituted and substituted Cp rings exposes that the two Cp rings are essentially parallel to each other.

The molecular structure is stabilized by weak intramolecular C—H···O interactions and the crystal packing is stabilized by a weak intermolecular C—H···O interaction. The interactions C3—H3···O3 generates an S(6) graph set motif and C13—H13···O2 generates an S(7) graph set motif (Bernstein *et al.*, 1995).

### Experimental

A mixture of 2-ferrocenylidene-2(*H*)-indene-1,2-dione (20 mmol) and sarcosine (30 mmol) and acenapthylene-1,2-dione (30 mmol) were refluxed in benzene for 20 h and the solvent was removed under reduced pressure. The crude product was subjected to column chromatography to get the pure product. Chloroform and methanol (1:1) solvent mixture was used for the crystallization under slow evaporation method.

### Refinement

H atoms were positioned geometrically and refined using riding model, with C—H = 0.93 Å and *U*_iso_(H) = 1.2*U*_eq_(C) for aromatic C—H, C—H = 0.98 Å and *U*_iso_(H) = 1.2*U*_eq_(C) for CH, C—H = 0.97 Å and *U*_iso_(H) = 1.2*U*_eq_(C) for CH_2_, and C—H = 0.96 Å and *U*_iso_(H) = 1.5*U*_eq_(C) for CH_3_. The components of the anisotropic displacement parameters in direction of the bond of C30, C31, C32 and C34 were restrained to be equal within an effective standard deviation of 0.001, using the DELU command in *SHELXL97* (Sheldrick, 2008).

### Crystal data

**Table:**

[Fe(C_5_H_5_)(C_29_H_20_NO_3_)]	*F*(000) = 1144
*M**_r_* = 551.40	*D*_x_ = 1.438 Mg m^−^^3^
Monoclinic, *P*2_1_/*c*	Mo *K*α radiation, λ = 0.71073 Å
Hall symbol: -P 2ybc	Cell parameters from 9589 reflections
*a* = 12.5511 (10) Å	θ = 2.2–28.7°
*b* = 10.8633 (9) Å	µ = 0.63 mm^−^^1^
*c* = 19.2099 (16) Å	*T* = 293 K
β = 103.432 (2)°	Block, colourless
*V* = 2547.6 (4) Å^3^	0.25 × 0.20 × 0.15 mm
*Z* = 4	

### Data collection

**Table:**

Bruker Kappa APEXII CCD diffractometer	6771 independent reflections
Radiation source: fine-focus sealed tube	5179 reflections with *I* > 2σ(*I*)
graphite	*R*_int_ = 0.031
Detector resolution: 0 pixels mm^-1^	θ_max_ = 29.0°, θ_min_ = 2.2°
φ and ω scans	*h* = −17→17
Absorption correction: multi-scan (*SADABS*; Sheldrick, 1996)	*k* = −14→14
*T*_min_ = 0.860, *T*_max_ = 0.911	*l* = −26→26
30734 measured reflections	

### Refinement

**Table:**

Refinement on *F*^2^	Primary atom site location: structure-invariant direct methods
Least-squares matrix: full	Secondary atom site location: difference Fourier map
*R*[*F*^2^ > 2σ(*F*^2^)] = 0.040	Hydrogen site location: inferred from neighbouring sites
*wR*(*F*^2^) = 0.124	H-atom parameters constrained
*S* = 1.02	*w* = 1/[σ^2^(*F*_o_^2^) + (0.0687*P*)^2^ + 0.7386*P*] where *P* = (*F*_o_^2^ + 2*F*_c_^2^)/3
6771 reflections	(Δ/σ)_max_ = 0.007
353 parameters	Δρ_max_ = 0.49 e Å^−^^3^
5 restraints	Δρ_min_ = −0.42 e Å^−^^3^

### Fractional atomic coordinates and isotropic or equivalent isotropic displacement parameters (Å^2^)

**Table:**

	*x*	*y*	*z*	*U*_iso_*/*U*_eq_	
C1	0.26851 (13)	0.32057 (15)	0.96632 (9)	0.0302 (3)	
C2	0.22528 (13)	0.22624 (14)	0.90588 (9)	0.0292 (3)	
C3	0.33376 (13)	0.19055 (16)	0.88341 (10)	0.0340 (4)	
H3	0.3493	0.2559	0.8520	0.041*	
C4	0.42067 (15)	0.1988 (2)	0.95383 (11)	0.0453 (5)	
H4A	0.4450	0.1171	0.9709	0.054*	
H4B	0.4835	0.2453	0.9472	0.054*	
C5	0.29193 (15)	0.45044 (16)	0.93572 (10)	0.0360 (4)	
C6	0.23280 (15)	0.54421 (16)	0.96773 (10)	0.0386 (4)	
C7	0.23105 (19)	0.67074 (18)	0.96368 (12)	0.0508 (5)	
H7	0.2661	0.7123	0.9330	0.061*	
C8	0.1748 (2)	0.7348 (2)	1.00733 (14)	0.0597 (6)	
H8	0.1727	0.8203	1.0052	0.072*	
C9	0.12269 (18)	0.6759 (2)	1.05296 (13)	0.0542 (5)	
H9	0.0864	0.7222	1.0810	0.065*	
C10	0.12281 (15)	0.54626 (19)	1.05833 (10)	0.0414 (4)	
C11	0.07709 (17)	0.4733 (2)	1.10488 (11)	0.0476 (5)	
H11	0.0369	0.5101	1.1343	0.057*	
C12	0.09210 (17)	0.3485 (2)	1.10665 (10)	0.0479 (5)	
H12	0.0617	0.3020	1.1378	0.057*	
C13	0.15220 (16)	0.28747 (18)	1.06292 (10)	0.0418 (4)	
H13	0.1628	0.2028	1.0664	0.050*	
C14	0.19421 (13)	0.35462 (15)	1.01581 (9)	0.0321 (3)	
C15	0.17929 (15)	0.48339 (15)	1.01399 (10)	0.0340 (4)	
C16	0.13789 (14)	0.27455 (16)	0.84251 (9)	0.0365 (4)	
C17	0.04269 (13)	0.19062 (16)	0.82985 (9)	0.0333 (3)	
C18	−0.05434 (16)	0.1963 (2)	0.77759 (11)	0.0457 (5)	
H18	−0.0663	0.2577	0.7429	0.055*	
C19	−0.13250 (17)	0.1074 (2)	0.77898 (13)	0.0557 (6)	
H19	−0.1978	0.1081	0.7441	0.067*	
C20	−0.11559 (18)	0.0177 (2)	0.83102 (14)	0.0560 (6)	
H20	−0.1703	−0.0401	0.8310	0.067*	
C21	−0.01942 (17)	0.01133 (18)	0.88323 (12)	0.0459 (5)	
H21	−0.0084	−0.0495	0.9182	0.055*	
C22	0.06043 (13)	0.09933 (15)	0.88157 (9)	0.0321 (3)	
C23	0.16939 (13)	0.11364 (14)	0.93040 (9)	0.0297 (3)	
C24	0.44333 (17)	0.3360 (2)	1.05712 (11)	0.0503 (5)	
H24A	0.4762	0.3974	1.0328	0.075*	
H24B	0.4996	0.2850	1.0854	0.075*	
H24C	0.4032	0.3754	1.0878	0.075*	
C25	0.32820 (14)	0.07159 (16)	0.84323 (10)	0.0343 (4)	
C26	0.34818 (15)	−0.05036 (17)	0.87150 (11)	0.0391 (4)	
H26	0.3681	−0.0714	0.9197	0.047*	
C27	0.33237 (16)	−0.13339 (19)	0.81320 (12)	0.0491 (5)	
H27	0.3406	−0.2184	0.8166	0.059*	
C28	0.30209 (18)	−0.0657 (2)	0.74947 (12)	0.0555 (6)	
H28	0.2866	−0.0981	0.7034	0.067*	
C29	0.29919 (16)	0.0608 (2)	0.76739 (11)	0.0460 (5)	
H29	0.2814	0.1256	0.7351	0.055*	
C30	0.5872 (2)	0.0968 (3)	0.8343 (2)	0.0886 (8)	
H30	0.5913	0.1709	0.8591	0.106*	
C31	0.60864 (19)	−0.0238 (4)	0.86344 (15)	0.0864 (8)	
H31	0.6298	−0.0450	0.9116	0.104*	
C32	0.5917 (2)	−0.1012 (3)	0.8066 (3)	0.0876 (9)	
H32	0.6000	−0.1863	0.8098	0.105*	
C33	0.5625 (3)	−0.0405 (5)	0.7471 (2)	0.0943 (11)	
H33	0.5464	−0.0756	0.7017	0.113*	
C34	0.5589 (2)	0.0793 (4)	0.7605 (2)	0.0915 (10)	
H34	0.5405	0.1411	0.7264	0.110*	
N1	0.36930 (11)	0.26055 (14)	1.00483 (8)	0.0358 (3)	
O1	0.14436 (15)	0.36625 (16)	0.80913 (10)	0.0759 (6)	
O2	0.20805 (11)	0.04640 (12)	0.97995 (7)	0.0436 (3)	
O3	0.35663 (13)	0.46876 (13)	0.89941 (8)	0.0510 (4)	
Fe1	0.45199 (2)	−0.00912 (2)	0.806961 (13)	0.03531 (9)	

### Atomic displacement parameters (Å^2^)

**Table:**

	*U*^11^	*U*^22^	*U*^33^	*U*^12^	*U*^13^	*U*^23^
C1	0.0306 (8)	0.0266 (7)	0.0349 (8)	−0.0055 (6)	0.0108 (6)	−0.0020 (6)
C2	0.0278 (7)	0.0257 (7)	0.0342 (8)	−0.0027 (6)	0.0078 (6)	0.0010 (6)
C3	0.0289 (8)	0.0335 (8)	0.0415 (9)	−0.0044 (6)	0.0117 (7)	−0.0050 (7)
C4	0.0284 (8)	0.0521 (11)	0.0529 (11)	−0.0009 (8)	0.0045 (8)	−0.0171 (9)
C5	0.0391 (9)	0.0308 (8)	0.0393 (9)	−0.0104 (7)	0.0116 (7)	−0.0027 (7)
C6	0.0423 (10)	0.0289 (8)	0.0449 (10)	−0.0046 (7)	0.0108 (8)	−0.0014 (7)
C7	0.0611 (13)	0.0298 (9)	0.0626 (13)	−0.0047 (9)	0.0169 (11)	0.0026 (9)
C8	0.0671 (15)	0.0317 (10)	0.0778 (16)	0.0063 (10)	0.0115 (13)	−0.0067 (10)
C9	0.0543 (12)	0.0457 (11)	0.0622 (13)	0.0101 (10)	0.0125 (11)	−0.0164 (10)
C10	0.0364 (9)	0.0442 (10)	0.0423 (10)	0.0023 (8)	0.0066 (8)	−0.0095 (8)
C11	0.0419 (10)	0.0632 (13)	0.0402 (10)	−0.0003 (9)	0.0149 (8)	−0.0129 (9)
C12	0.0488 (11)	0.0607 (13)	0.0382 (10)	−0.0118 (9)	0.0185 (9)	−0.0017 (9)
C13	0.0462 (10)	0.0399 (10)	0.0417 (10)	−0.0075 (8)	0.0150 (8)	0.0011 (8)
C14	0.0306 (8)	0.0330 (8)	0.0334 (8)	−0.0050 (6)	0.0086 (7)	−0.0032 (6)
C15	0.0339 (8)	0.0328 (8)	0.0348 (9)	−0.0019 (7)	0.0071 (7)	−0.0031 (6)
C16	0.0365 (9)	0.0344 (9)	0.0380 (9)	−0.0007 (7)	0.0071 (7)	0.0040 (7)
C17	0.0310 (8)	0.0348 (8)	0.0341 (8)	0.0018 (7)	0.0075 (7)	−0.0043 (7)
C18	0.0399 (10)	0.0514 (11)	0.0414 (10)	0.0067 (8)	0.0004 (8)	−0.0048 (9)
C19	0.0355 (10)	0.0652 (14)	0.0583 (13)	−0.0017 (10)	−0.0056 (9)	−0.0146 (11)
C20	0.0367 (10)	0.0562 (13)	0.0714 (16)	−0.0166 (9)	0.0050 (10)	−0.0085 (11)
C21	0.0401 (10)	0.0406 (10)	0.0562 (12)	−0.0121 (8)	0.0097 (9)	−0.0005 (8)
C22	0.0296 (8)	0.0305 (8)	0.0362 (8)	−0.0026 (6)	0.0079 (7)	−0.0049 (6)
C23	0.0296 (7)	0.0246 (7)	0.0353 (8)	−0.0028 (6)	0.0084 (6)	−0.0017 (6)
C24	0.0435 (11)	0.0518 (11)	0.0492 (11)	−0.0111 (9)	−0.0020 (9)	−0.0137 (9)
C25	0.0280 (8)	0.0350 (8)	0.0391 (9)	0.0007 (7)	0.0064 (7)	−0.0053 (7)
C26	0.0355 (9)	0.0358 (9)	0.0488 (10)	−0.0018 (7)	0.0155 (8)	−0.0017 (8)
C27	0.0401 (10)	0.0396 (10)	0.0680 (14)	−0.0024 (8)	0.0135 (10)	−0.0158 (10)
C28	0.0451 (11)	0.0648 (14)	0.0506 (12)	0.0027 (10)	−0.0008 (9)	−0.0256 (11)
C29	0.0389 (10)	0.0539 (12)	0.0401 (10)	0.0084 (9)	−0.0010 (8)	−0.0064 (9)
C30	0.0441 (13)	0.0844 (13)	0.148 (2)	−0.0253 (12)	0.0450 (16)	−0.0605 (15)
C31	0.0293 (10)	0.179 (2)	0.0490 (12)	−0.0047 (16)	0.0056 (10)	0.0330 (12)
C32	0.0522 (15)	0.0571 (12)	0.166 (3)	0.0133 (12)	0.0508 (19)	0.0076 (16)
C33	0.077 (2)	0.141 (3)	0.081 (2)	−0.015 (2)	0.0515 (19)	−0.029 (2)
C34	0.0549 (15)	0.114 (3)	0.1110 (17)	0.0018 (16)	0.0296 (16)	0.0689 (17)
N1	0.0302 (7)	0.0357 (7)	0.0395 (8)	−0.0041 (6)	0.0041 (6)	−0.0068 (6)
O1	0.0702 (11)	0.0640 (11)	0.0797 (12)	−0.0189 (9)	−0.0106 (9)	0.0421 (9)
O2	0.0469 (7)	0.0342 (6)	0.0447 (7)	−0.0056 (6)	0.0003 (6)	0.0102 (6)
O3	0.0604 (9)	0.0424 (7)	0.0608 (9)	−0.0197 (7)	0.0351 (8)	−0.0063 (6)
Fe1	0.03380 (15)	0.04039 (16)	0.03252 (15)	0.00359 (10)	0.00929 (10)	−0.00137 (10)

### Geometric parameters (Å, °)

**Table:**

C1—N1	1.462 (2)	C20—C21	1.380 (3)
C1—C14	1.524 (2)	C20—H20	0.9300
C1—C2	1.549 (2)	C21—C22	1.391 (2)
C1—C5	1.582 (2)	C21—H21	0.9300
C2—C16	1.530 (2)	C22—C23	1.476 (2)
C2—C23	1.537 (2)	C23—O2	1.209 (2)
C2—C3	1.569 (2)	C24—N1	1.453 (2)
C3—C25	1.499 (2)	C24—H24A	0.9600
C3—C4	1.530 (2)	C24—H24B	0.9600
C3—H3	0.9800	C24—H24C	0.9600
C4—N1	1.456 (2)	C25—C29	1.422 (3)
C4—H4A	0.9700	C25—C26	1.432 (3)
C4—H4B	0.9700	C25—Fe1	2.0424 (17)
C5—O3	1.204 (2)	C26—C27	1.416 (3)
C5—C6	1.476 (3)	C26—Fe1	2.0459 (19)
C6—C7	1.377 (3)	C26—H26	0.9300
C6—C15	1.398 (3)	C27—C28	1.403 (3)
C7—C8	1.401 (3)	C27—Fe1	2.043 (2)
C7—H7	0.9300	C27—H27	0.9300
C8—C9	1.368 (3)	C28—C29	1.419 (3)
C8—H8	0.9300	C28—Fe1	2.042 (2)
C9—C10	1.412 (3)	C28—H28	0.9300
C9—H9	0.9300	C29—Fe1	2.0386 (19)
C10—C15	1.405 (3)	C29—H29	0.9300
C10—C11	1.412 (3)	C30—C34	1.391 (5)
C11—C12	1.368 (3)	C30—C31	1.426 (5)
C11—H11	0.9300	C30—Fe1	2.017 (2)
C12—C13	1.417 (3)	C30—H30	0.9300
C12—H12	0.9300	C31—C32	1.356 (5)
C13—C14	1.361 (2)	C31—Fe1	2.019 (2)
C13—H13	0.9300	C31—H31	0.9300
C14—C15	1.411 (2)	C32—C33	1.296 (5)
C16—O1	1.198 (2)	C32—Fe1	2.021 (2)
C16—C17	1.478 (2)	C32—H32	0.9300
C17—C22	1.385 (2)	C33—C34	1.330 (5)
C17—C18	1.388 (2)	C33—Fe1	2.026 (3)
C18—C19	1.381 (3)	C33—H33	0.9300
C18—H18	0.9300	C34—Fe1	2.018 (3)
C19—C20	1.377 (3)	C34—H34	0.9300
C19—H19	0.9300		
			
N1—C1—C14	112.06 (14)	Fe1—C26—H26	126.6
N1—C1—C2	101.27 (13)	C28—C27—C26	108.42 (18)
C14—C1—C2	118.01 (13)	C28—C27—Fe1	69.89 (13)
N1—C1—C5	111.84 (13)	C26—C27—Fe1	69.85 (11)
C14—C1—C5	102.05 (13)	C28—C27—H27	125.8
C2—C1—C5	111.98 (13)	C26—C27—H27	125.8
C16—C2—C23	102.89 (13)	Fe1—C27—H27	126.1
C16—C2—C1	115.53 (14)	C27—C28—C29	108.25 (18)
C23—C2—C1	113.69 (13)	C27—C28—Fe1	69.95 (12)
C16—C2—C3	111.93 (14)	C29—C28—Fe1	69.51 (11)
C23—C2—C3	111.99 (13)	C27—C28—H28	125.9
C1—C2—C3	101.21 (12)	C29—C28—H28	125.9
C25—C3—C4	116.36 (15)	Fe1—C28—H28	126.2
C25—C3—C2	114.52 (13)	C28—C29—C25	108.25 (19)
C4—C3—C2	103.20 (14)	C28—C29—Fe1	69.80 (11)
C25—C3—H3	107.4	C25—C29—Fe1	69.75 (10)
C4—C3—H3	107.4	C28—C29—H29	125.9
C2—C3—H3	107.4	C25—C29—H29	125.9
N1—C4—C3	106.59 (14)	Fe1—C29—H29	126.2
N1—C4—H4A	110.4	C34—C30—C31	104.7 (3)
C3—C4—H4A	110.4	C34—C30—Fe1	69.89 (16)
N1—C4—H4B	110.4	C31—C30—Fe1	69.39 (15)
C3—C4—H4B	110.4	C34—C30—H30	127.6
H4A—C4—H4B	108.6	C31—C30—H30	127.6
O3—C5—C6	126.83 (17)	Fe1—C30—H30	124.7
O3—C5—C1	124.85 (17)	C32—C31—C30	105.9 (3)
C6—C5—C1	107.81 (14)	C32—C31—Fe1	70.46 (16)
C7—C6—C15	120.27 (19)	C30—C31—Fe1	69.22 (14)
C7—C6—C5	131.96 (19)	C32—C31—H31	127.1
C15—C6—C5	107.51 (15)	C30—C31—H31	127.1
C6—C7—C8	117.8 (2)	Fe1—C31—H31	124.9
C6—C7—H7	121.1	C33—C32—C31	110.7 (3)
C8—C7—H7	121.1	C33—C32—Fe1	71.53 (18)
C9—C8—C7	122.3 (2)	C31—C32—Fe1	70.32 (16)
C9—C8—H8	118.9	C33—C32—H32	124.6
C7—C8—H8	118.9	C31—C32—H32	124.6
C8—C9—C10	121.4 (2)	Fe1—C32—H32	125.1
C8—C9—H9	119.3	C32—C33—C34	110.0 (3)
C10—C9—H9	119.3	C32—C33—Fe1	71.11 (17)
C15—C10—C9	115.6 (2)	C34—C33—Fe1	70.49 (18)
C15—C10—C11	116.59 (18)	C32—C33—H33	125.0
C9—C10—C11	127.7 (2)	C34—C33—H33	125.0
C12—C11—C10	119.84 (19)	Fe1—C33—H33	125.0
C12—C11—H11	120.1	C33—C34—C30	108.7 (3)
C10—C11—H11	120.1	C33—C34—Fe1	71.12 (17)
C11—C12—C13	122.57 (19)	C30—C34—Fe1	69.79 (16)
C11—C12—H12	118.7	C33—C34—H34	125.7
C13—C12—H12	118.7	C30—C34—H34	125.7
C14—C13—C12	119.02 (18)	Fe1—C34—H34	125.0
C14—C13—H13	120.5	C24—N1—C4	114.41 (15)
C12—C13—H13	120.5	C24—N1—C1	115.95 (15)
C13—C14—C15	118.58 (17)	C4—N1—C1	109.36 (14)
C13—C14—C1	132.28 (16)	C30—Fe1—C34	40.32 (15)
C15—C14—C1	108.80 (14)	C30—Fe1—C31	41.39 (14)
C6—C15—C10	122.69 (17)	C34—Fe1—C31	67.09 (12)
C6—C15—C14	113.81 (16)	C30—Fe1—C32	66.72 (13)
C10—C15—C14	123.34 (18)	C34—Fe1—C32	64.36 (14)
O1—C16—C17	125.32 (17)	C31—Fe1—C32	39.22 (14)
O1—C16—C2	125.89 (17)	C30—Fe1—C33	66.29 (14)
C17—C16—C2	108.79 (14)	C34—Fe1—C33	38.39 (16)
C22—C17—C18	121.22 (17)	C31—Fe1—C33	65.30 (14)
C22—C17—C16	109.57 (15)	C32—Fe1—C33	37.36 (15)
C18—C17—C16	129.18 (17)	C30—Fe1—C29	123.12 (12)
C19—C18—C17	117.58 (19)	C34—Fe1—C29	108.80 (11)
C19—C18—H18	121.2	C31—Fe1—C29	160.75 (14)
C17—C18—H18	121.2	C32—Fe1—C29	157.71 (15)
C20—C19—C18	121.23 (19)	C33—Fe1—C29	124.05 (14)
C20—C19—H19	119.4	C30—Fe1—C25	109.11 (9)
C18—C19—H19	119.4	C34—Fe1—C25	125.66 (14)
C19—C20—C21	121.63 (19)	C31—Fe1—C25	125.00 (11)
C19—C20—H20	119.2	C32—Fe1—C25	160.66 (14)
C21—C20—H20	119.2	C33—Fe1—C25	160.29 (15)
C20—C21—C22	117.52 (19)	C29—Fe1—C25	40.79 (7)
C20—C21—H21	121.2	C30—Fe1—C28	157.86 (15)
C22—C21—H21	121.2	C34—Fe1—C28	121.85 (13)
C17—C22—C21	120.81 (17)	C31—Fe1—C28	157.88 (15)
C17—C22—C23	110.57 (14)	C32—Fe1—C28	122.70 (13)
C21—C22—C23	128.61 (17)	C33—Fe1—C28	107.98 (12)
O2—C23—C22	125.46 (15)	C29—Fe1—C28	40.70 (9)
O2—C23—C2	126.44 (15)	C25—Fe1—C28	68.61 (8)
C22—C23—C2	108.08 (13)	C30—Fe1—C27	161.32 (14)
N1—C24—H24A	109.5	C34—Fe1—C27	155.96 (15)
N1—C24—H24B	109.5	C31—Fe1—C27	123.59 (12)
H24A—C24—H24B	109.5	C32—Fe1—C27	108.86 (11)
N1—C24—H24C	109.5	C33—Fe1—C27	122.10 (14)
H24A—C24—H24C	109.5	C29—Fe1—C27	68.14 (9)
H24B—C24—H24C	109.5	C25—Fe1—C27	68.65 (7)
C29—C25—C26	107.04 (16)	C28—Fe1—C27	40.16 (10)
C29—C25—C3	124.67 (17)	C30—Fe1—C26	125.69 (12)
C26—C25—C3	128.28 (17)	C34—Fe1—C26	162.60 (15)
C29—C25—Fe1	69.46 (11)	C31—Fe1—C26	109.55 (9)
C26—C25—Fe1	69.64 (10)	C32—Fe1—C26	124.70 (13)
C3—C25—Fe1	126.71 (12)	C33—Fe1—C26	157.45 (16)
C27—C26—C25	108.03 (18)	C29—Fe1—C26	68.36 (8)
C27—C26—Fe1	69.64 (12)	C25—Fe1—C26	40.99 (7)
C25—C26—Fe1	69.37 (10)	C28—Fe1—C26	68.00 (9)
C27—C26—H26	126.0	C27—Fe1—C26	40.51 (8)
C25—C26—H26	126.0		
			
N1—C1—C2—C16	−163.27 (14)	C33—C34—Fe1—C31	78.6 (2)
C14—C1—C2—C16	74.06 (19)	C30—C34—Fe1—C31	−40.5 (2)
C5—C1—C2—C16	−43.96 (19)	C33—C34—Fe1—C32	35.5 (2)
N1—C1—C2—C23	78.07 (15)	C30—C34—Fe1—C32	−83.6 (2)
C14—C1—C2—C23	−44.6 (2)	C30—C34—Fe1—C33	−119.1 (3)
C5—C1—C2—C23	−162.62 (13)	C33—C34—Fe1—C29	−121.5 (2)
N1—C1—C2—C3	−42.18 (15)	C30—C34—Fe1—C29	119.39 (19)
C14—C1—C2—C3	−164.85 (14)	C33—C34—Fe1—C25	−163.8 (2)
C5—C1—C2—C3	77.13 (16)	C30—C34—Fe1—C25	77.1 (2)
C16—C2—C3—C25	−75.73 (18)	C33—C34—Fe1—C28	−78.5 (2)
C23—C2—C3—C25	39.2 (2)	C30—C34—Fe1—C28	162.39 (18)
C1—C2—C3—C25	160.67 (14)	C33—C34—Fe1—C27	−43.2 (4)
C16—C2—C3—C4	156.81 (15)	C30—C34—Fe1—C27	−162.3 (2)
C23—C2—C3—C4	−88.24 (16)	C33—C34—Fe1—C26	161.0 (3)
C1—C2—C3—C4	33.21 (16)	C30—C34—Fe1—C26	41.9 (4)
C25—C3—C4—N1	−138.43 (16)	C32—C31—Fe1—C30	−116.4 (3)
C2—C3—C4—N1	−12.14 (19)	C32—C31—Fe1—C34	−77.0 (2)
N1—C1—C5—O3	52.2 (2)	C30—C31—Fe1—C34	39.4 (2)
C14—C1—C5—O3	172.19 (18)	C30—C31—Fe1—C32	116.4 (3)
C2—C1—C5—O3	−60.7 (2)	C32—C31—Fe1—C33	−34.9 (2)
N1—C1—C5—C6	−120.07 (16)	C30—C31—Fe1—C33	81.5 (2)
C14—C1—C5—C6	−0.12 (18)	C32—C31—Fe1—C29	−158.4 (3)
C2—C1—C5—C6	127.04 (15)	C30—C31—Fe1—C29	−42.0 (4)
O3—C5—C6—C7	2.6 (4)	C32—C31—Fe1—C25	164.54 (18)
C1—C5—C6—C7	174.7 (2)	C30—C31—Fe1—C25	−79.04 (19)
O3—C5—C6—C15	−171.30 (19)	C32—C31—Fe1—C28	41.8 (3)
C1—C5—C6—C15	0.8 (2)	C30—C31—Fe1—C28	158.2 (3)
C15—C6—C7—C8	0.3 (3)	C32—C31—Fe1—C27	78.5 (2)
C5—C6—C7—C8	−173.0 (2)	C30—C31—Fe1—C27	−165.12 (16)
C6—C7—C8—C9	0.1 (4)	C32—C31—Fe1—C26	121.36 (19)
C7—C8—C9—C10	−0.1 (4)	C30—C31—Fe1—C26	−122.23 (17)
C8—C9—C10—C15	−0.1 (3)	C33—C32—Fe1—C30	−80.9 (3)
C8—C9—C10—C11	177.0 (2)	C31—C32—Fe1—C30	40.14 (19)
C15—C10—C11—C12	2.1 (3)	C33—C32—Fe1—C34	−36.5 (2)
C9—C10—C11—C12	−175.0 (2)	C31—C32—Fe1—C34	84.6 (2)
C10—C11—C12—C13	−0.3 (3)	C33—C32—Fe1—C31	−121.1 (3)
C11—C12—C13—C14	−1.9 (3)	C31—C32—Fe1—C33	121.1 (3)
C12—C13—C14—C15	2.1 (3)	C33—C32—Fe1—C29	40.3 (4)
C12—C13—C14—C1	174.51 (17)	C31—C32—Fe1—C29	161.4 (2)
N1—C1—C14—C13	−53.8 (2)	C33—C32—Fe1—C25	−162.3 (3)
C2—C1—C14—C13	63.2 (3)	C31—C32—Fe1—C25	−41.2 (4)
C5—C1—C14—C13	−173.61 (18)	C33—C32—Fe1—C28	76.3 (3)
N1—C1—C14—C15	119.20 (15)	C31—C32—Fe1—C28	−162.64 (18)
C2—C1—C14—C15	−123.78 (16)	C33—C32—Fe1—C27	118.5 (2)
C5—C1—C14—C15	−0.61 (17)	C31—C32—Fe1—C27	−120.40 (19)
C7—C6—C15—C10	−0.5 (3)	C33—C32—Fe1—C26	160.8 (2)
C5—C6—C15—C10	174.26 (17)	C31—C32—Fe1—C26	−78.2 (2)
C7—C6—C15—C14	−176.06 (18)	C32—C33—Fe1—C30	82.2 (3)
C5—C6—C15—C14	−1.3 (2)	C34—C33—Fe1—C30	−38.1 (2)
C9—C10—C15—C6	0.4 (3)	C32—C33—Fe1—C34	120.3 (3)
C11—C10—C15—C6	−177.01 (18)	C32—C33—Fe1—C31	36.6 (2)
C9—C10—C15—C14	175.54 (18)	C34—C33—Fe1—C31	−83.7 (2)
C11—C10—C15—C14	−1.9 (3)	C34—C33—Fe1—C32	−120.3 (3)
C13—C14—C15—C6	175.32 (17)	C32—C33—Fe1—C29	−162.8 (2)
C1—C14—C15—C6	1.2 (2)	C34—C33—Fe1—C29	76.9 (2)
C13—C14—C15—C10	−0.2 (3)	C32—C33—Fe1—C25	162.6 (3)
C1—C14—C15—C10	−174.29 (16)	C34—C33—Fe1—C25	42.3 (4)
C23—C2—C16—O1	175.8 (2)	C32—C33—Fe1—C28	−120.7 (2)
C1—C2—C16—O1	51.3 (3)	C34—C33—Fe1—C28	118.9 (2)
C3—C2—C16—O1	−63.8 (3)	C32—C33—Fe1—C27	−78.9 (3)
C23—C2—C16—C17	−3.16 (18)	C34—C33—Fe1—C27	160.79 (19)
C1—C2—C16—C17	−127.64 (15)	C32—C33—Fe1—C26	−44.9 (4)
C3—C2—C16—C17	117.25 (15)	C34—C33—Fe1—C26	−165.3 (2)
O1—C16—C17—C22	−175.7 (2)	C28—C29—Fe1—C30	159.64 (17)
C2—C16—C17—C22	3.26 (19)	C25—C29—Fe1—C30	−80.92 (17)
O1—C16—C17—C18	2.4 (3)	C28—C29—Fe1—C34	117.3 (2)
C2—C16—C17—C18	−178.70 (17)	C25—C29—Fe1—C34	−123.24 (18)
C22—C17—C18—C19	−0.1 (3)	C28—C29—Fe1—C31	−168.5 (3)
C16—C17—C18—C19	−177.94 (19)	C25—C29—Fe1—C31	−49.0 (3)
C17—C18—C19—C20	1.1 (3)	C28—C29—Fe1—C32	49.3 (3)
C18—C19—C20—C21	−1.1 (4)	C25—C29—Fe1—C32	168.8 (3)
C19—C20—C21—C22	0.1 (3)	C28—C29—Fe1—C33	77.6 (2)
C18—C17—C22—C21	−0.9 (3)	C25—C29—Fe1—C33	−162.95 (17)
C16—C17—C22—C21	177.32 (17)	C28—C29—Fe1—C25	−119.44 (19)
C18—C17—C22—C23	179.88 (16)	C25—C29—Fe1—C28	119.44 (19)
C16—C17—C22—C23	−1.90 (19)	C28—C29—Fe1—C27	−37.22 (14)
C20—C21—C22—C17	0.9 (3)	C25—C29—Fe1—C27	82.22 (12)
C20—C21—C22—C23	179.97 (19)	C28—C29—Fe1—C26	−80.99 (14)
C17—C22—C23—O2	−178.74 (17)	C25—C29—Fe1—C26	38.45 (11)
C21—C22—C23—O2	2.1 (3)	C29—C25—Fe1—C30	118.92 (17)
C17—C22—C23—C2	−0.19 (18)	C26—C25—Fe1—C30	−122.85 (17)
C21—C22—C23—C2	−179.33 (18)	C3—C25—Fe1—C30	0.3 (2)
C16—C2—C23—O2	−179.41 (17)	C29—C25—Fe1—C34	77.04 (18)
C1—C2—C23—O2	−53.7 (2)	C26—C25—Fe1—C34	−164.74 (16)
C3—C2—C23—O2	60.2 (2)	C3—C25—Fe1—C34	−41.5 (2)
C16—C2—C23—C22	2.06 (17)	C29—C25—Fe1—C31	162.31 (16)
C1—C2—C23—C22	127.74 (14)	C26—C25—Fe1—C31	−79.46 (17)
C3—C2—C23—C22	−118.31 (15)	C3—C25—Fe1—C31	43.7 (2)
C4—C3—C25—C29	−147.00 (18)	C29—C25—Fe1—C32	−167.1 (3)
C2—C3—C25—C29	92.6 (2)	C26—C25—Fe1—C32	−48.9 (3)
C4—C3—C25—C26	34.1 (3)	C3—C25—Fe1—C32	74.3 (4)
C2—C3—C25—C26	−86.3 (2)	C29—C25—Fe1—C33	46.1 (4)
C4—C3—C25—Fe1	−57.9 (2)	C26—C25—Fe1—C33	164.3 (3)
C2—C3—C25—Fe1	−178.34 (12)	C3—C25—Fe1—C33	−72.5 (4)
C29—C25—C26—C27	0.6 (2)	C26—C25—Fe1—C29	118.22 (16)
C3—C25—C26—C27	179.61 (17)	C3—C25—Fe1—C29	−118.6 (2)
Fe1—C25—C26—C27	−59.11 (13)	C29—C25—Fe1—C28	−37.58 (13)
C29—C25—C26—Fe1	59.66 (13)	C26—C25—Fe1—C28	80.64 (13)
C3—C25—C26—Fe1	−121.29 (17)	C3—C25—Fe1—C28	−156.16 (19)
C25—C26—C27—C28	−0.5 (2)	C29—C25—Fe1—C27	−80.85 (13)
Fe1—C26—C27—C28	−59.45 (15)	C26—C25—Fe1—C27	37.37 (12)
C25—C26—C27—Fe1	58.94 (12)	C3—C25—Fe1—C27	160.57 (18)
C26—C27—C28—C29	0.3 (2)	C29—C25—Fe1—C26	−118.22 (16)
Fe1—C27—C28—C29	−59.15 (15)	C3—C25—Fe1—C26	123.2 (2)
C26—C27—C28—Fe1	59.43 (14)	C27—C28—Fe1—C30	−170.1 (2)
C27—C28—C29—C25	0.1 (2)	C29—C28—Fe1—C30	−50.6 (3)
Fe1—C28—C29—C25	−59.35 (13)	C27—C28—Fe1—C34	158.58 (18)
C27—C28—C29—Fe1	59.42 (15)	C29—C28—Fe1—C34	−81.9 (2)
C26—C25—C29—C28	−0.4 (2)	C27—C28—Fe1—C31	50.4 (3)
C3—C25—C29—C28	−179.48 (17)	C29—C28—Fe1—C31	169.9 (2)
Fe1—C25—C29—C28	59.38 (14)	C27—C28—Fe1—C32	80.5 (2)
C26—C25—C29—Fe1	−59.77 (12)	C29—C28—Fe1—C32	−160.01 (17)
C3—C25—C29—Fe1	121.14 (17)	C27—C28—Fe1—C33	118.80 (19)
C34—C30—C31—C32	−0.1 (3)	C29—C28—Fe1—C33	−121.71 (19)
Fe1—C30—C31—C32	61.34 (18)	C27—C28—Fe1—C29	−119.49 (18)
C34—C30—C31—Fe1	−61.40 (18)	C27—C28—Fe1—C25	−81.83 (12)
C30—C31—C32—C33	−0.2 (3)	C29—C28—Fe1—C25	37.66 (12)
Fe1—C31—C32—C33	60.3 (2)	C29—C28—Fe1—C27	119.49 (18)
C30—C31—C32—Fe1	−60.51 (17)	C27—C28—Fe1—C26	−37.56 (12)
C31—C32—C33—C34	0.4 (4)	C29—C28—Fe1—C26	81.93 (13)
Fe1—C32—C33—C34	60.0 (2)	C28—C27—Fe1—C30	168.4 (3)
C31—C32—C33—Fe1	−59.6 (2)	C26—C27—Fe1—C30	48.8 (3)
C32—C33—C34—C30	−0.4 (4)	C28—C27—Fe1—C34	−49.6 (3)
Fe1—C33—C34—C30	59.9 (2)	C26—C27—Fe1—C34	−169.1 (2)
C32—C33—C34—Fe1	−60.4 (2)	C28—C27—Fe1—C31	−159.61 (16)
C31—C30—C34—C33	0.3 (3)	C26—C27—Fe1—C31	80.86 (18)
Fe1—C30—C34—C33	−60.8 (2)	C28—C27—Fe1—C32	−118.71 (18)
C31—C30—C34—Fe1	61.06 (17)	C26—C27—Fe1—C32	121.76 (18)
C3—C4—N1—C24	−147.53 (17)	C28—C27—Fe1—C33	−79.7 (2)
C3—C4—N1—C1	−15.5 (2)	C26—C27—Fe1—C33	160.75 (18)
C14—C1—N1—C24	−65.40 (19)	C28—C27—Fe1—C29	37.70 (12)
C2—C1—N1—C24	167.91 (15)	C26—C27—Fe1—C29	−81.83 (13)
C5—C1—N1—C24	48.5 (2)	C28—C27—Fe1—C25	81.72 (13)
C14—C1—N1—C4	163.43 (14)	C26—C27—Fe1—C25	−37.81 (11)
C2—C1—N1—C4	36.75 (17)	C26—C27—Fe1—C28	−119.53 (17)
C5—C1—N1—C4	−82.66 (17)	C28—C27—Fe1—C26	119.53 (17)
C31—C30—Fe1—C34	−115.3 (3)	C27—C26—Fe1—C30	−162.73 (17)
C34—C30—Fe1—C31	115.3 (3)	C25—C26—Fe1—C30	77.76 (17)
C34—C30—Fe1—C32	77.2 (2)	C27—C26—Fe1—C34	165.1 (3)
C31—C30—Fe1—C32	−38.06 (19)	C25—C26—Fe1—C34	45.6 (4)
C34—C30—Fe1—C33	36.4 (2)	C27—C26—Fe1—C31	−119.22 (18)
C31—C30—Fe1—C33	−78.9 (2)	C25—C26—Fe1—C31	121.28 (16)
C34—C30—Fe1—C29	−80.0 (2)	C27—C26—Fe1—C32	−78.16 (19)
C31—C30—Fe1—C29	164.72 (16)	C25—C26—Fe1—C32	162.34 (16)
C34—C30—Fe1—C25	−123.0 (2)	C27—C26—Fe1—C33	−46.7 (3)
C31—C30—Fe1—C25	121.67 (17)	C25—C26—Fe1—C33	−166.2 (3)
C34—C30—Fe1—C28	−43.0 (3)	C27—C26—Fe1—C29	81.24 (13)
C31—C30—Fe1—C28	−158.3 (2)	C25—C26—Fe1—C29	−38.26 (11)
C34—C30—Fe1—C27	157.2 (3)	C27—C26—Fe1—C25	119.50 (16)
C31—C30—Fe1—C27	41.9 (4)	C27—C26—Fe1—C28	37.25 (13)
C34—C30—Fe1—C26	−165.77 (18)	C25—C26—Fe1—C28	−82.26 (12)
C31—C30—Fe1—C26	78.94 (19)	C25—C26—Fe1—C27	−119.50 (16)
C33—C34—Fe1—C30	119.1 (3)		

### Hydrogen-bond geometry (Å, °)

**Table:**

*D*—H···*A*	*D*—H	H···*A*	*D*···*A*	*D*—H···*A*
C3—H3···O3	0.98	2.48	3.045 (2)	116
C13—H13···O2	0.93	2.53	3.226 (2)	132
C8—H8···O2^i^	0.93	2.56	3.465 (6)	164

Symmetry codes: (i) *x*, *y*+1, *z*.
